# A novel, cheap and easy preparing selective medium for isolation of *Pythium* species

**DOI:** 10.1186/s13568-025-01946-x

**Published:** 2025-10-13

**Authors:** Sahar A. Moharam, Amna M. Sadek, Hani M. A. Abdelzaher, Mahmoud A. Shoulkamy, Hussam H. Arafat

**Affiliations:** Department of Botany and Microbiology, Faculty of Science, Minia University, El-Minia, 61519 Egypt

**Keywords:** Mycelial growth, Oospore production, *Pythium*, Selective medium, Zoospore formation

## Abstract

Isolation of *Pythium* and *Globisporangium* (Chromista, Oomycota) from their natural sources is very difficult due to the heavy contamination with other microbes, which hinders obtaining them in pure form. This study aims to develop a new, cheap and safe selective medium to isolate *Pythium* and *Globisporangium* species from their natural sources, despite the previous presence of more than 3 types of selective media including: VP_3_ (Vancomycin + PCNB + Penicillin + Pimaricin), NARM (Nystatin + Ampicillin + Rifampicin + Miconazole), and NARF (Nystatin + Ampicillin + Rifampicin + Fluazinam) used to isolate Oomycota. The elevated expense of antibiotics, along with alerts regarding potential contamination, toxicity, and tumor proliferation, renders them undesirable. Safe and inexpensive antibiotics from pharmaceutical sources were used with fixed formulations and concentrations. A selective medium was developed, called FANS (Fluconazole + Ampicillin + Nystatin + Sulbactam) with the following concentrations (1200 mg + 500 mg + 100,000 IU + 250 mg) respectively/L Corn Meal Agar promotes only the growth and formation of reproductive structures of *Pythium* and *Globisporangium*. On the other hand, these antibiotics completely inhibited the growth of other contaminated fungi and bacteria. Mycelial growth, zoospore formation, and oospore production for (*Pythium aphanidermatum*,* Pythium oligandrum*, and *Globisporangium ultimum* var. *ultimum*) were highest in FANS, followed by NARM, then NARF, while VP_3_ gave the lowest results compared to the control sample. It can be concluded that the use of FANS selective medium made the process of *Pythium* isolation easier, safer, and less expensive for worldwide researchers especially in developing and low-income countries.

## Introduction

Since the beginning of the study of mycology, scientists have been searching for effective ways to isolate specific fungi from a large intermingling community of genera and species. Therefore, selective culture media have been developed to isolate genera or even species of fungi from their selected sources. Yuriko et al. (2008) developed a selective media for the isolation of yeasts and filamentous fungi from the sputum of adult patients with cystic fibrosis (CF). Zhang et al. (2021) manufactured a selective flamingo medium for the isolation of *Aspergillus fumigatus*. In summary, the library of previous studies is filled with many types of selective media for isolating specific groups of fungi, including: *Armillaria* selective medium, *Aspergillus* selective medium, *Cylindrocarpon* selective medium, *Fuasarium* selective medium, *Gaeumannomyces* selective medium, *Pyrenochaeta* selective medium, *Verticillium* selective medium, *Rhizoctonia* selective medium ….etc (https://wiki.bugwood.org/Diagnosticians_cookbook, 2024).

Since the discovery of genus *Pythium* by the biologist Pringsheim in 1858, that belongs to the family Pythiaceae within the order Pythiales, phylum Oomycota and kingdom Chromista, there have been many attempts to isolate these Oomycota (Pringsheim 1858; Burr and Stanghellini 1973; Ali-Shtayeh et al. 1986; Jeffers and Martin 1986; Oudemans 1999; Morita and Tojo 2007; Tojo 2017). Isolation in the past depended on culturing infected parts of plants or water in normal nutrient media to isolate fungi in general. Some of these selective media have been created but have been found to have some problems. Triple P (Pimaricin/Penicillin/Polymyxin) had a problem in which some *Pythium* spp. did not grow on it, presumably as a result of it inhibiting sporangium germination (Eckert and Tsao 1962). We found that all of these media have obstacles that prevent them from being easily and cheaply obtained and available in various regions around the world. For example, some media ingredients have been found to contain carcinogenic chemicals that some countries prohibit their use. Some of these media heavily depend on the fungicide pentachloronitrobenzene (PCNB), however commercial pesticide products are not available since PCNB contains the carcinogenic contaminant hexachlorobenzene (HCB) (Choudhury et al. 1987). Moreover, the food sanitation regulation in numerous nations, prohibits the commercial sale of crude pimaricin, another essential component of many previously mentioned selective media (Rencüzoğullari et al. 2009; Rasgele and Kaymak 2013; Tojo 2017). It is worth noting that one of the most important reasons for the lack of research in the field of *Pythium*, especially in poor countries, is the high price of antifungal and antibacterial drugs in addition to the difficulty of obtaining them.

All selective media such as VP_3_, NARM and NARF developed for the isolation of *Pythium* have been tested for their effectiveness on other fungi and bacteria and are not effective against *Pythium*. It is worth noting that the ineffectiveness of these selective media was confirmed only towards the mycelial growth of the *Pythium*, while their effectiveness towards the production of asexual and sexual spores was not tested. This is because it has been proven that some antibiotics inhibit the production of asexual and sexual structures and do not inhibit the production of mycelial growth (Yang et al. 2024).

This study aims to produce a selective nutrient medium for isolating *Pythium* spp., consisting of a selected group of antifungal and antibacterial antibiotics that do not affect the isolation of *Pythium* spp. It’s worth noting that all antibiotics, both antifungal and bacterial, used in FANS Medium are derived from pharmaceutical sources used to treat certain human diseases. Since the repeated use of antibiotics can affect the production of asexual and sexual structures in *Pythium*, they are used only during the isolation phase from natural sources. All subsequent experiments are then conducted on media that do not contain these antibiotics. There may be concerns about their broader pharmacological or environmental implications. It is important to note, however, that these compounds were only used for in vitro research purposes in controlled, small-scale laboratory settings.

The FANS drugs are cheap and accessible to researchers globally. In addition to its technical importance, the development of such affordable microbiological tools is a crucial component of circular bioeconomy and sustainable biotechnology initiatives, especially when it comes to disease control in environments with limited resources, such as developing countries. The genus *Pythium* is a prominent pathogen that affect a broad host range, including plants, humans and animals such as dogs and horses. *Pythium insidiosum*, specifically, causes pythiosis, a sever infectious disease seen in tropical and subtropical regions, affecting humans and animals. Clinically, it can cause vascular, ocular, cutaneous, subcutaneous, and gastrointestinal infections, and in dogs gastrointestinal pythiosis with a high mortality rate (Hu et al. 2024; Krajaejun et al. 2024; Nguyen et al. 2022). In animals such as dogs and horses, the infection acquiring from water sources containing the pathogen, arising as cutaneous and subcutaneous lesions, often called swamp cancer or leeches in equines (Radice et al. 2024; Today’s Veterinary Nurse 2025).

In addition, *Pythium* species cause significant root rot and damping-off diseases affecting global agricultural production, resulting in economic losses ranging from 20 to 40% of crop yields annually (Lamichhane et al. 2017; Russo et al. 2023; Özgen and Kilic 2025). They are soilborne pathogens thrive in wet conditions, with spores spread through contaminated soil, water, and tools (Wang et al. 2020). As reliance on chemical control strategies becomes less sustainable due to environmental concerns and resistance development (Thambugala et al. 2020; Kipngeno et al. 2015), there is a growing demand for low-cost, environmentally friendly microbial tools. Thus, prompting research for alternatives including microbial biocontrol agents and waste valorization techniques (Friends of the Earth 2024; Russo et al. 2023). Developing selective media for the isolation and study of plant pathogens, research on sustainable control methods directly related to biotechnological innovations focused on sustainable plant protection and circular bioeconomy practices (Jayaraj et al. 2006; Russo et al. 2023). *Pythium irregulare* has been successfully cultivated using food-industry by-products such as spent brewery yeast and expired orange juice, significantly reducing costs and enabling efficient EPA (eicosapentaenoic acid) production (Russo et al. 2023).

## Materials and methods

### Isolation of Pythium spp. from rhizosphere of crop plants (Fig. 1)

#### Sample collection

*Pythium* spp. that were isolated and tested in this study included *Globisporangium ultimum* Trow var. *ultimum*, *Pythium aphanidermatum* Edson, and *Pythium oligandrum* Dreschler, obtained from the rhizosphere of potatoes (*Solanum tuberosum* L.) grown in a farm in the west gate of Minia University, Egypt, in December 2023; from soybeans (*Glycine max.* L.) grown in several agricultural farms in Matai city, Minia, Egypt, in August 2024; and from eggplant (*Solanum melongena* L.) grown in the Botanical Farm of the Faculty of Science, Minia University, Egypt, in August 2024, respectively. While this study focuses on using FANS medium to isolate *Pythium spp.* and *G. ultimum* var. *ultimum* from rhizosphere of crop plants, the FANS selective medium was also used to evaluate samples from geographically distinct field sites, greenhouse-grown vegetable crops: potatoes (*Solanum tuberosum* L.), soybeans (*Glycine max.* L.), eggplant (*Solanum melongena* L.),okra (*Abelmoschus esculentus* L.), lettuce (*Lactuca sativa* L.), sweet pepper (*Capsicum annuum* L.), maize (*Zea mays* L.), and chili pepper (*Capsicum* spp. L.), and fish farms to fully assess its performance in a variety of environmental and agricultural contexts.

#### Isolation process

To isolate *Pythium* spp. and *Globisporangium ultimum* var. *ultimum* from the rhizosphere of various plants, two methods were used in this study: A direct soil inoculation method, in this method; a suitable amount of soil from the rhizosphere of (*Solanum tuberosum* L., *Glycine max.* L. and *Solanum melongena* L.) was placed in Petri-dishes containing selective medium appropriate for capturing only those *Pythium* spp. Then, the plates were incubated at 25 °C in the dark until colonies appeared (Abdelzaher et al. 2020).

Baiting technique method according to (Harvey 1925; Lodhi et al. 2004; Watanabe et al. 2008; Abdelzaher et al. 2020). In this method; sterile swabs were used to collect samples from plant rhizospheres; approximately 10 cm of roots were cut and placed in a flask and shaking vigorously. Approximately 10–15 ml of the suspension was transferred into sterile sample collection containers, and several sterilized (3 × 14) long pieces of *Echinochloa colonum* L. leaves were added, and then incubated in the dark for 5 days at 25 °C. They were then transferred to selective medium by placing them in the four corners of a 9 cm Petri-dish and incubated at 25 °C in the dark for 2–3 days until hyphal threads observed.

#### Purification

Each colony was purified by placing small plug from actively growing hyphal tip on corn meal agar (CMA) and incubating at 25 °C until growth reached just before the edge of the plate. An inoculum of *Pythium* growth was taken from the edge of the colony and placed in the center of a Petri-dish containing 2% water agar (WA). It was incubated until growth reached two-thirds of the dish. Small, sterile pieces of *E. colonum* L. leaves were placed at the edge of the colony and incubated for 24-h at 20 °C until hyphae colonized the used grass leaf. The grass leaf pieces colonized by *Pythium* hyphae were placed in a Petri-dish filled with sterile distilled water and incubated at 20 °C for 24-h. The production of asexual structures and swimming zoospores was then monitored. The sample was examined every 24-h to observe the formation of sexual structures, which usually appear after the period of asexual reproduction which requires between 2 and 7 days.

#### Identification of the isolated Pythium species

The *Pythium* spp. were identified on the basis of morphology as these species have clear asexual and sexual structures. Asexual and sexual structures are considered the most important criteria for identifying *Pythium* spp. Zoosporangia and their shapes, the production of swimming zoospores, and non-spore-producing asexual structures are among the most important units of asexual reproduction and are included in the identification of these fungi. Meanwhile, shapes, numbers of antheridia and their method of attachment to the oogonia, as well as the shape of the oogonia, thickness and thorns of their walls, and number of oospores and thickness of their walls are among the most important units of sexual reproduction and are also included in the identification of these microbes (Plaats-Niterink 1981; Dick 1990; Abdelzaher et al. 2020). They were then photographed, and figures were made for each species using an OPTIKA compound microscope (ZEISS, Carl Zeiss AG, West Germany).

#### Preservation of isolates

Short-term storage for *Pythium* isolates was maintained on cornmeal agar slants at 5 °C, with subculturing onto fresh media every 3 months as described by Robertson (1980). For long-term preservation, *Pythium* isolates were cryopreserved by culturing on carbon filter paper strips, soaking them in 10% glycerol, subjecting them to an initial freezing step at − 20 °C, as described by Lee (2023). The three studied strains were deposited at the culture collection, Ain Shams University (CCASU), held by the Faculty of Pharmacy at Ain Shams University under specific codes: *Pythium aphanidermatum* (CCASU-2025-F15), *Pythium oligandrum* (CCASU-2025-F16), and *Globisporangium ultimum* var. *ultimum* (CCASU-2025-F17).

**Fig. 1:**
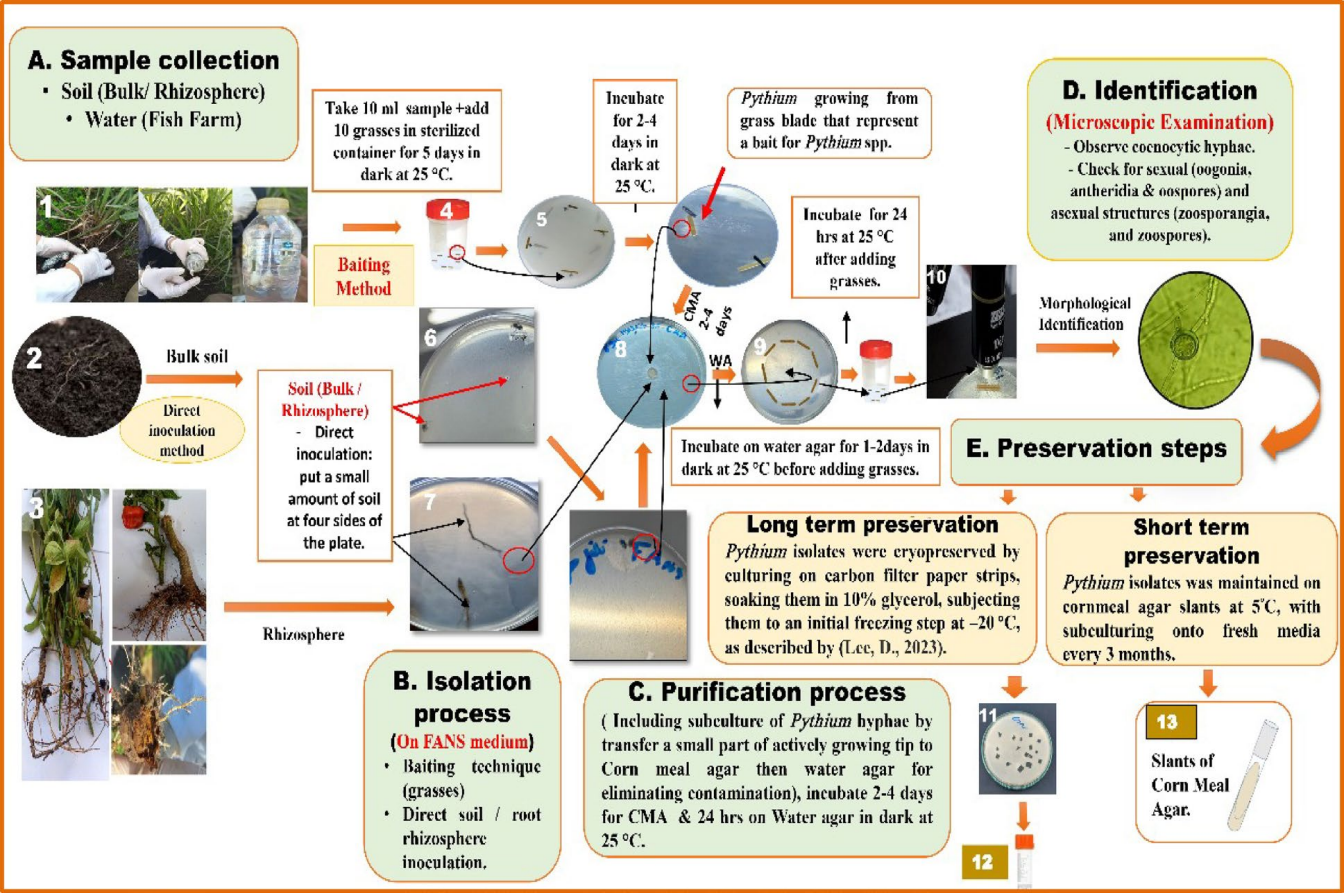
Schematic workflow illustrating the protocol for *Pythium* isolation from crop plants (soil, plant rhizosphere), and water samples, including **A** sample collection (1–3), **B** isolation process (4–7), **C** purification process (8, 9), **D** morphological identification (10) and **E** preservation (11–13)

To make a comparison between the selective medium proposed by this study (FANS medium) and three well-known types of selective media (VP_3_, NARF and NARM), these media were prepared in the following ways:

### Previous media formula

A 20 g/L of Corn Meal Agar (CMA), Marca: BBL as the base medium for NARF and NARM as well as FANS selective media.

#### Antibiotic of (Vancomycin + PCNB + Penicillin + Pimaricin) VP_3_

A Five mg, pimaricin (Sigma-Aldrich) dissolved in 1 ml of ethanol, 75 mg vancomycin (C) dissolved in 1 ml of distilled water (DW), 50 mg penicillin (Sigma-Aldrich) dissolved in 1 ml of DW, 100 mg PCNB (Sigma, Aldrich) dissolved in 1 ml DW (Ali-Shtayeh et al. 1986; with some modification of the basal medium only).

#### Antibiotic of (Nystatin + Ampicillin + Rifampicin + Fluazinam) NARF

A Fifty mg, nystatin (Sigma-Aldrich, St. Louis, MO, USA) dissolved in 1 ml of ethanol, 250 mg ampicillin (Sigma-Aldrich) dissolved in 1 ml of distilled water (DW), 10 mg rifampicin (Sigma-Aldrich) dissolved in 1 ml of dimethyl sulfoxide (DMSO), 0.5 mg fluazinam (Froncide Wettable Powder^®^, Nippon Soda, Tokyo, Japan) dissolved in 1 ml of 0.1% sterile water agar (Tojo 2017).

#### Antibiotics of (Nystatin + Ampicillin + Rifampicin + Miconazole) NARM

A 250 mg/L ampicillin (Sigma-Aldrich) dissolved in 1 ml DW, 10 mg/L rifampicin (Sigma-Aldrich) dissolved in 1 ml DMSO, 10 mg/L nystatin (Sigma-Aldrich) dissolved in 1 ml ethanol, 1 mg/L (Sigma-Aldrich) miconazole diluted in 1 ml of DMSO. After autoclaving and cooling to 50 °C, each antibiotic was added to the basal medium. The mixture was thoroughly mixed using a magnetic stirrer (VWR^®^, VWR, USA), and 10 ml of the medium was then transferred into each Petri-dishes with a diameter of 9 cm (Tojo 2017).

#### Preparation of the new selective medium, Fluconazole-Ampicillin-Nystatin-Sulbactam (FANS)

A 500 mg ampicillin dissolved in 2.5 ml DW, 250 mg sulbactam dissolved in 2.5 ml DW, 100,000 IU/L nystatin dissolved in 16 ml 70% ethanol, 1200 mg fluconazole in 8 ml of DMSO. After autoclaving and cooling to 50 °C, each antibiotic was added to the 1 L basal medium. The mixture was thoroughly mixed using a magnetic stirrer, and 10 ml of the medium was then transferred into Petri-dishes with a diameter of 9 cm. The following Table 1 shows the price differences (in USD) between the antibiotics used in FANS medium and those used in previously established media (VP_3_, NARM, and NARF).

**Table 1:** Cost comparison of antibiotics used in FANS medium versus previous selective media

FANS medium		Other media	
The compound	Average price of antibiotics in the Egyptian market ($)	The compound	Sigma-Aldrich antibiotic prices ($)
Fluconazole (1 gm)	9.05	Miconazole (1 gm)	163.80
1200 mg/L	10.86		
Nystatin 20 ml (100,000 IU)	0.12	Nystatin 20 ml (100,000 IU)	30.71
30 ml (100,000 IU)	0.17	Natamycin (1 gm)	259.35
		Pimaricin (25 mg)	487.31
Ampicillin 1000 mg + Sulbactam 500 mg (5 ml)	= 1.03	Ampicillin (500 mg)	350.8
		Sulbactam500 mg	229.32
Ampicillin + Sulbactam (2.5 ml)	= 0.52		
Total price required for (1 L) of FANS	= 11.55		

Prices of May 2024, Annual inflation rate for medicine prices in Egypt is 12%

#### Selection of lethal concentration for contamination with Penicillium italicum Wehmer and Rhizopus stolonifer Vuillemin in FANS selection medium

In this experiment, different concentrations of antibacterial and antifungal agents were tested to reach the lowest lethal concentration for fungi and bacteria present in the culture medium and at the same time not to negatively affect *Pythium* spp. It was found that at concentrations that were effective on other fungi, growth of *P. italicum* and *R. stolonifer* appeared. Therefore, higher concentrations were tested until the lethal concentration for these two fungi was reached, while at the same time it was ineffective on the growth of *Pythium*. It is worth noting that the lethal concentration for *R. stolonifer* is different from the lethal concentration for *P. italicum*. It is worth noting that FANS medium has been tested for its inhibitory effect on other microorganisms that may cause contamination in natural environments (e.g., three bacterial species such as *Bacillus*,* Serratia* and *Staphylococcus* species). In addition, two other filamentous fungi like *Fusarium oxysporum* and *Trichoderma* sp.).

#### Effect of VP_3_, NARF, NARM and FANS selective media on mycelial growth of P. aphanidermatum, P. oligandrum and G. ultimum var. ultimum

Each *Pythium* sp. was inoculated to create mycelia for 10 days at 25 °C (Abdelzaher et al. 2020) in 250 ml Erlenmeyer flasks with 50 ml of Potato Dextrose Broth (PDB) in order to investigate the impact of adding antibiotics on mycelial growth. After that, mycelial mats were gathered in filter papers and dried in an oven set to 85 °C for 24-h to produce mycelial growth. Once the dry mats had cooled in a desiccator, they were weighed.

#### Effect of VP_3_, NARF, NARM and FANS selective media on zoospore formation of P. aphanidermatum and P. oligandrum

Since *Globisporangium ultimum* var. *ultimum* does not produce swimming spores, this experiment was conducted on *P. aphanidermatum* and *P. oligandrum* only. *Echinochloa colonum* L. grass blades were chopped into (3 × 14) mm pieces and autoclaved for 20 min at 121 °C. *Pythium aphanidermatum* and *P. oligandrum* were incubated on Petri-dishes with 2% water agar. After the fungal colonies attained a diameter of approximately 4 cm, the autoclaved *E. colonum* leaf blades were placed over each inoculum in contact with the actively growing margin, and incubated at 25 °C. Following 24-h incubation period, the colonized *E. colonum* leaf blades were moved to 7 cm-diam Petri-dishes, each of which contained 10 ml sterilized DW containing varying concentrations of the aforementioned antibiotics (for VP_3_, NARM, NARF and FANS). The plates were then incubated at 25 °C to determine the impact of the various antibiotics on the production of zoospores at various temperatures.

At various intervals, cultures were viewed under a microscope though the Petri-dish lid along the *E. colonum* leaf margin. At the apex of evacuation tubes, filamentous, inflated zoosporangia from *P. aphanidermatum* and *P. oligandrum* create vesicles. Every active vesicle contained zoospores that were prepared to release at a certain time.

When a vesicle is about to discharge, its zoospore differentiation indicates that it is active. The quantity of active zoosporangial vesicles along the leaf margin was counted using the (Elnaghy et al. 2010), approach, which was a modification of the Saleem and Dick method (Saleem and Dick 1990). For every treatment, a mean number of vesicles per millimeter were determined. A third set of partial data was collected for confirmation after the entire experiment was conducted twice with three replications.

#### Effect of VP_3_, NARF, NARM and FANS selective media on oospore production of P. aphanidermatum, P. oligandrum and G. ultimum var. ultimum

The *Pythium* spp. and *G. ultimum* var. *ultimum* were cultivated to develop oospores at various temperatures at 25 °C for three weeks in 100 ml Erlenmeyer flasks with 10 ml of V-8 juice (8-vegetable juice, supplemented with components of the tested selective media) in order to investigate the impact of temperature on oospore production. Mycelial mats were then chopped in a blender for three minutes to create oospore suspensions. To create a suspension of oospores that was comparatively free of hyphal fragments, the size of the sieve used to filter the resultant suspension was determined by the diameter of the oospores in addition to placing the suspension of oospores in the freezer for two days until the hyphae explode and only the oospores remain. Mature oospore counts were made and correlated with the criteria under examination.

### Statistical analysis

Unless otherwise noted, Minitab statistical software (version 12) was used to evaluate the data using one-way analysis of variance (one-way ANOVA).

## Results

### Isolation and identification of Pythium spp.

**Fig. 2:**
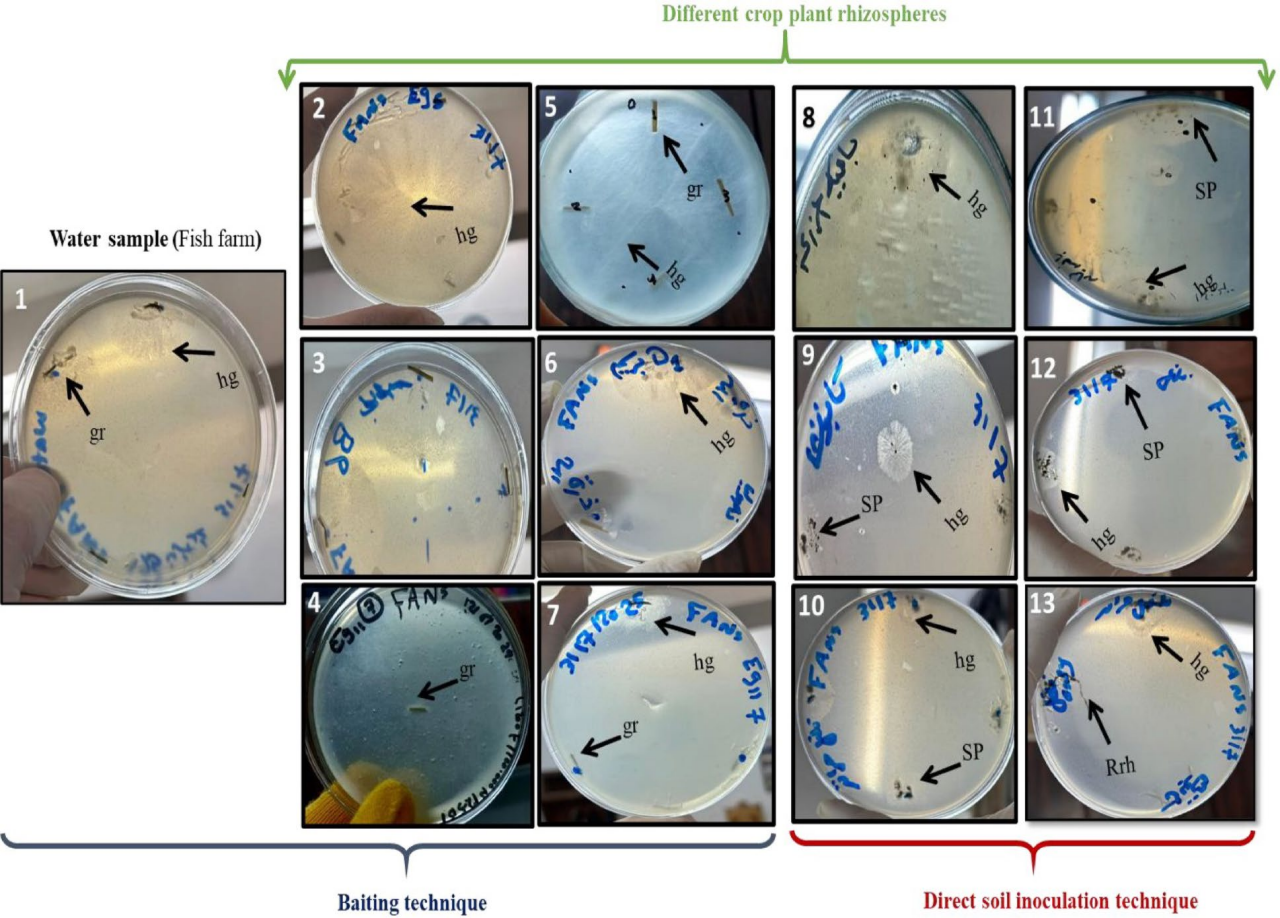
Isolation of *Pythium* species from various natural sources including: Fish farm (1) and different crop plant rhizospheres (2–13): potatoes (*Solanum tuberosum* L.), soybeans (*Glycine max.* L.), eggplant (*Solanum melongena* L.), okra (*Abelmoschus esculentus* L.), lettuce (*Lactuca sativa* L.), sweet pepper (*Capsicum annuum* L.), maize (*Zea mays* L.), and chili pepper (*Capsicum* spp. L.) using two techniques: Baiting technique (1–7) using grasses (*Echinochloa colonum* L.); Direct soil inoculation technique (8–13) on FANS medium at 25 °C for 5 days in dark. Note, (gr = grass, SP = Soil particles, hg = hyphal growth and Rrh = Root rhizosphere)

**Fig. 3:**
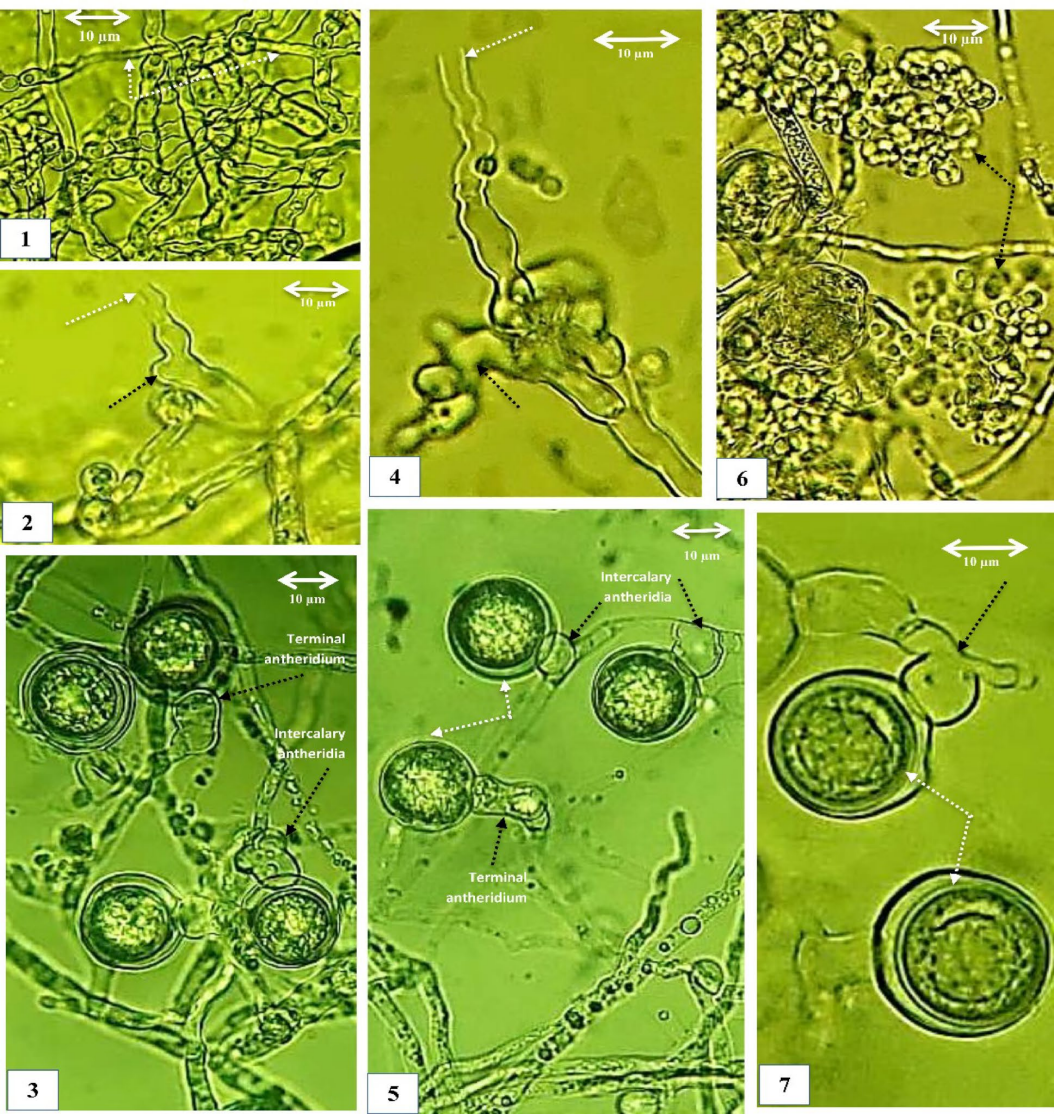
Morphology of *Pythium aphanidermatum* (CCASU-2025-F15). (1) Coenocytic hypha. (2, 4) Lobulated zoosporangia (black arrows). (2,4) Evacuation tubes (white arrows). 6. Clusters of encysted zoospores just emerging from a zoosporangium. (3, 5, 7) Diclinous antheridia mostly intercalary and sometimes terminal (black arrows) attached to terminal, smoothed and globose oogonia (white arrows). (3, 5, 7) Aplerotic oospores. Scale Bar on each picture equals 10 μm

**Fig. 4:**
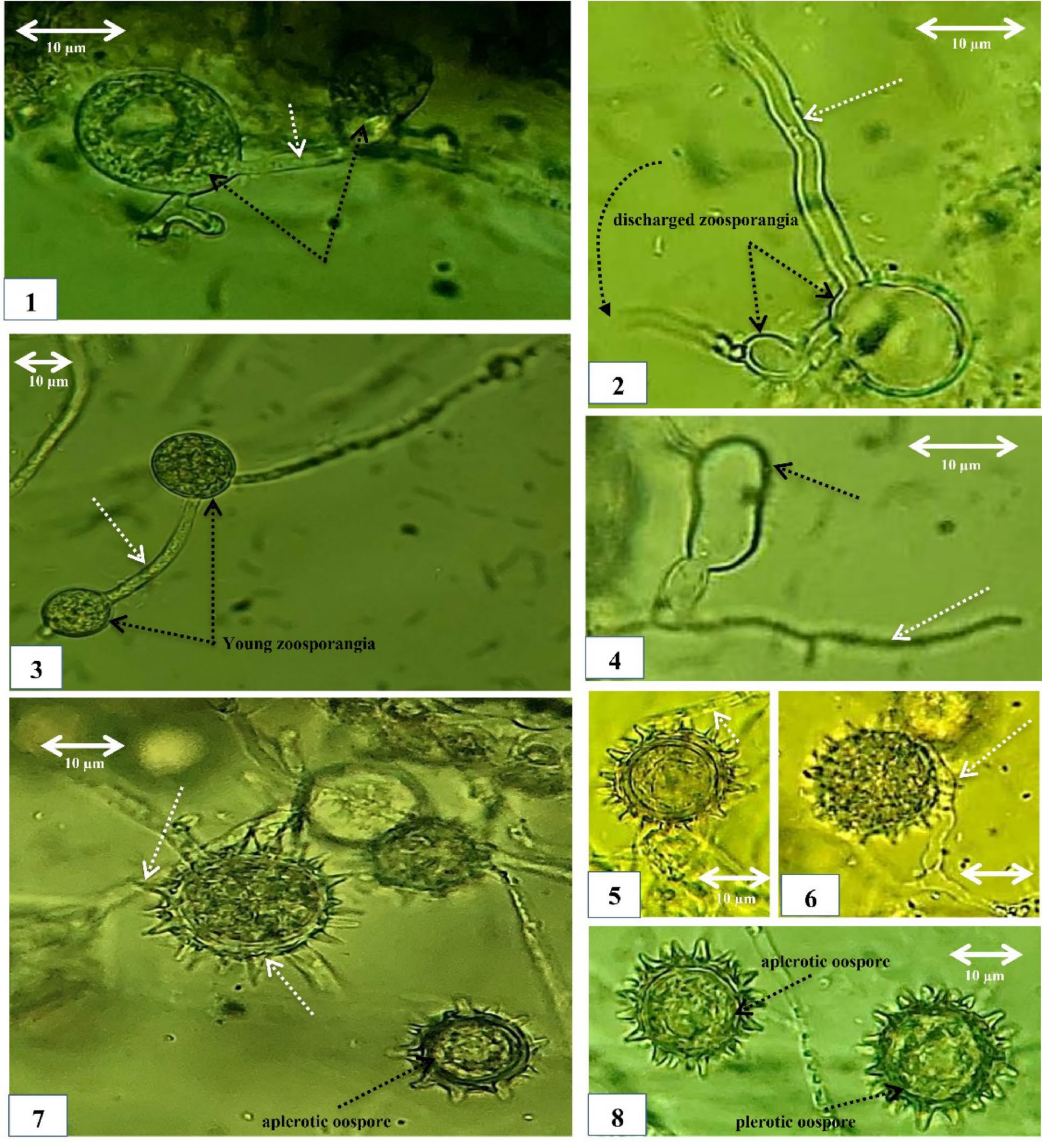
Morphology of *Pythium oligandrum* (CCASU-2025-F16). (1–4) Intercalary zoosporangia, forming irregular aggregates of one or more subglobose elements (black arrows) with connecting filamentous parts (white arrows). (2) Evacuation tube (curved arrow). (5,6) Young terminal oogonia encircled with thin antheridia (white arrows). (7) Spiny oogonia with spikes of sharp edges and a relatively broad base (white arrows). (7,8) Plerotic and aplerotic oospores. Scale bar on each picture equals 10 μm

**Fig. 5:**
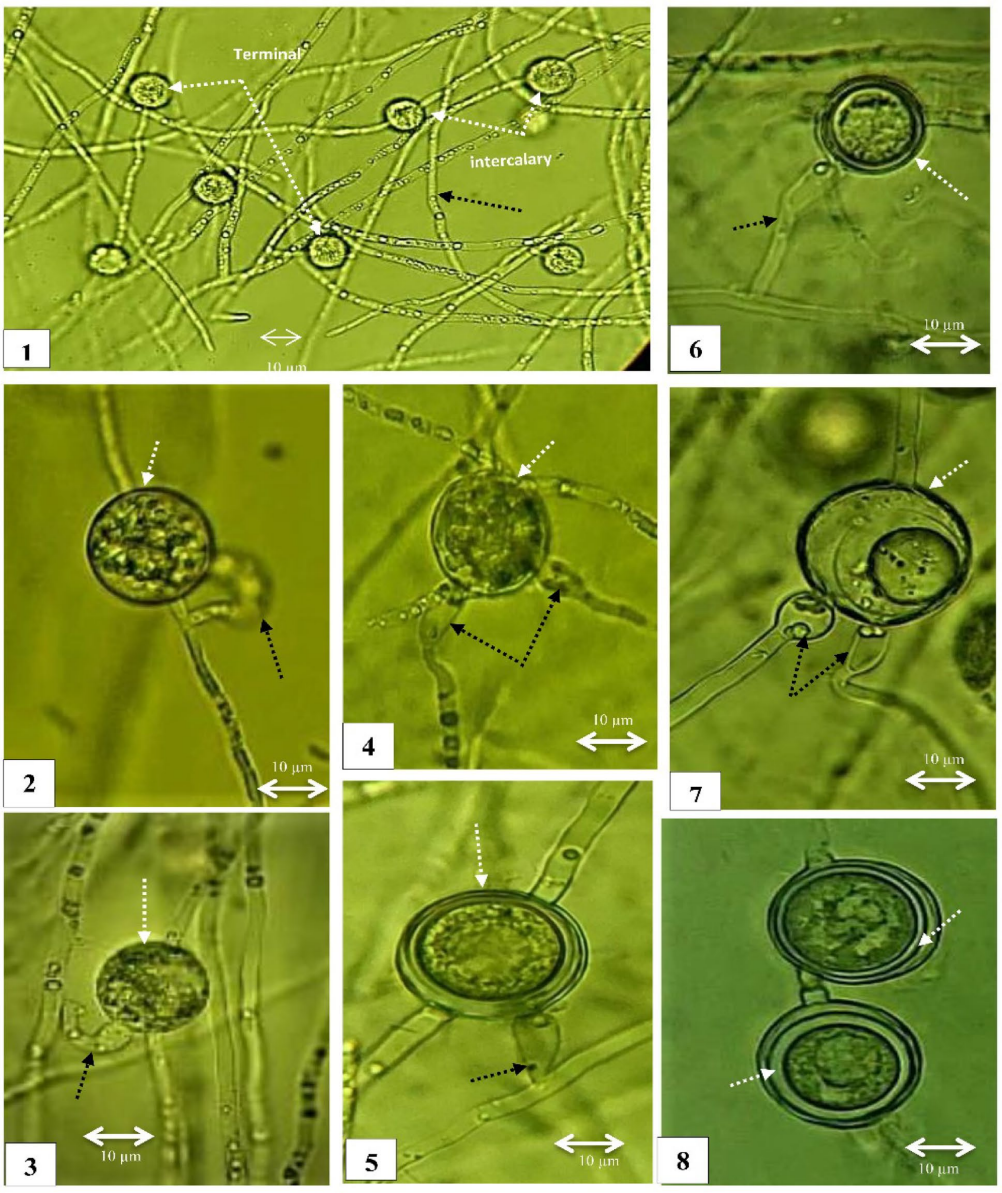
Morphology of *Globisporangium ultimum* var. *ultimum* (CCASU-2025-F17). (1) Coenocytic hyphae (black arrow) with multiple globose, intercalary and terminal hyphal swellings (white arrows). (2) Monoclinous antheridium (black arrow). (3,4,5,6,7) Diclinous (1–2) antheridia per oogonium (black arrows). (2,4,5) Intercalary oogonia (white arrows). (3,6,7) Terminal oogonia (white arrows). (8) Different thickness of aplerotic oospores. Scale bar on each picture equals 10 μm

*Pythium aphanidermatum* (isolate No. CCASU-2025-F15), *P. oligandrum* (CCASU-2025-F16), and *G. ultimum* var. *ultimum* (CCASU-2025-F17) were obtained from rhizosphere soil of soybean (*Glycine max*. L.) grown in an agricultural farm in Matai city, Minia, Egypt in August 2024; from the rhizosphere soil of eggplant grown in the Faculty of Science, Minia University, Egypt, in August 2024; and from the rhizosphere soil of potato (*Solanum tuberosum* L.) grown in a farm in the west gate of Minia University, Egypt, in December 2023, respectively. FANS selective medium proved its efficiency performance for selectively isolating *Pythium* spp. from geographically distinct field sites, greenhouse-grown vegetable crops, and fish farms, Minia Governorate, Egypt (Figs. 2, 3, 4, 5).

In addition to the original description of each species, morphological identification was carried out using the keys of (Middleton 1943; Waterhouse 1968; Plaats-Niterink 1981; Dick 1990).

### Lethal concentration selection for R. stolonifer and P. italicum contamination in FANS selection medium

Concentrations of antibacterial (500 mg ampicillin and 250 mg sulbactam) used in FANS selective medium inhibited bacteria and showed no contamination, bacterial colonies or bacteria adhering to the *Pythium* hyphae. As for antifungals, at low concentrations of the selective medium developed in this study, contamination with *P. italicum* and *R. stolonifer* was observed. For this reason, the concentrations of antifungals were increased until the lethal concentration for these two fungi was reached.

The concentration of 1200 mg of fluconazole was the lethal concentration for *P. italicum*, while the concentrations started from 300, 600 and 800 mg/L gave heavy, medium and light growth, respectively. In contrast, *R. stolonifer* was not affected by any of the concentrations tested Table 2.

**Table 2:** Effect of different concentrations of fluconazole on growth of *Penicillium italicum* and *Rhizopus stolonifer*

Conc. of fluconazole/L CMA*	300 mg	600 mg	800 mg	1200 mg
Mycelial growth *P. italicum*	+++	++	+	−
Mycelial growth *R. stolonifer*	+++	+++	+++	+++

+++ Heavy growth, ++ Medium growth, + Light growth, − No growth (lethal concentration)

The results are the average of three readings, and the entire experiment was repeated twice, with the third for final confirmation

**Fig. 6:**
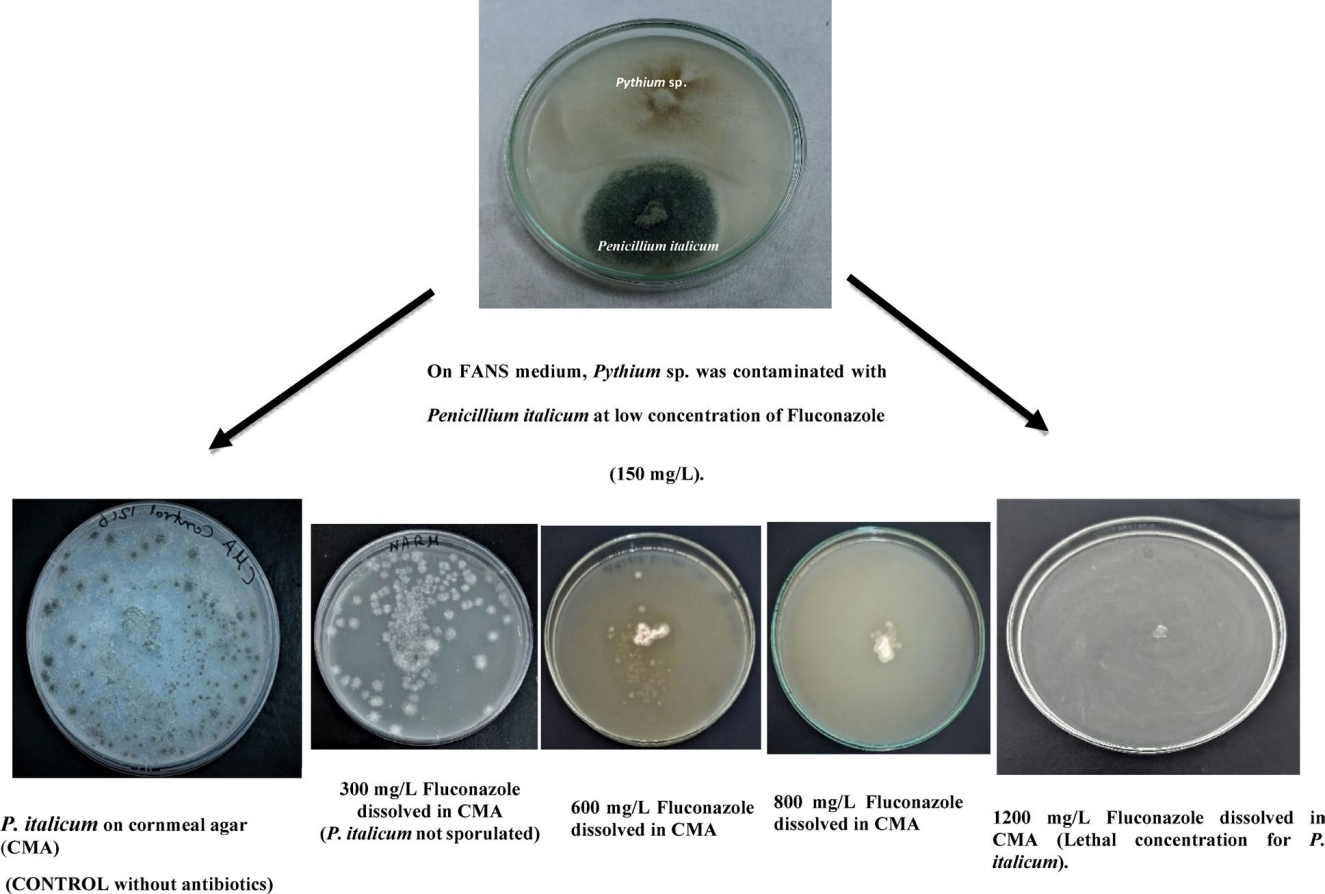
Sequential effect of fluconazole on fungal infection with *Penicillium italicum* until lethal concentration effect is reached

Since *R. stolonifer* was not affected by the applied concentrations of fluconazole, the effect of mycostatin was only tested on this fungus. A concentration of 100,000 IU/L of mycostatin did not give any growth of *R. stolonifer* compared to concentrations of 17,000, 34,000, 50,000, 67,000 and 84,000 IU/L which equivalent (3.86, 7.73, 11.36, 15.24, 19.09) mg/L respectively. which gave heavy, heavy, heavy, medium and light growth, respectively Table 3.

**Table 3:** Effect of different concentrations of Mycostatin on growth of *Rhizopus. Stolonifer*

Conc. of mycostatin/L CMA	17,000 IU*	34,000 IU*	50,000 IU*	67,000 IU*	84,000 IU*	100,000 IU*
Mycelial growth	+++	+++	+++	++	+	−

* International unit

+++ Heavy growth, ++ Medium growth, + Light growth, − No growth (lethal concentration)

The results are the average of three readings, and the entire experiment was repeated twice, with the third for final confirmation

**Fig. 7:**
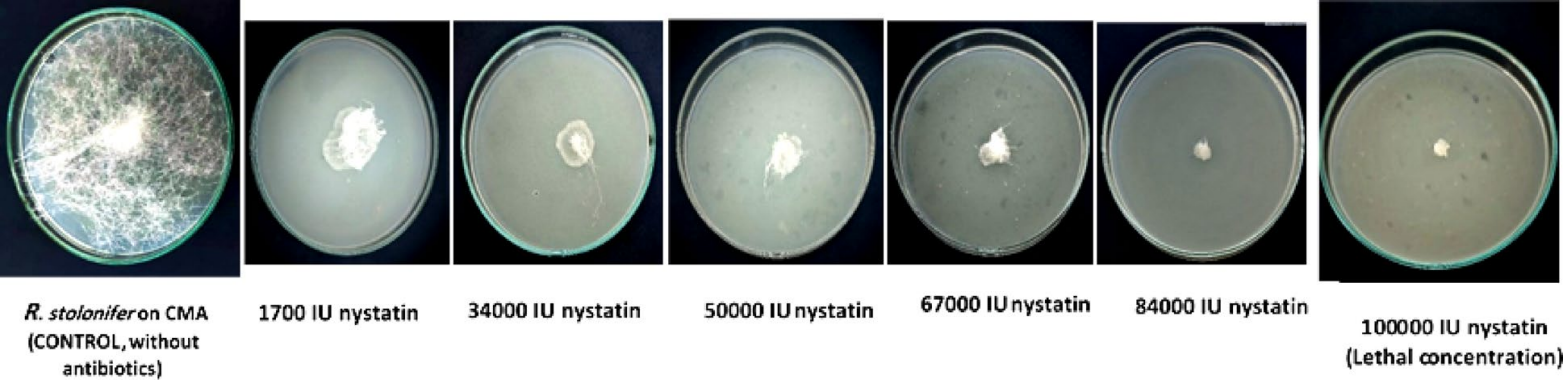
Sequential effects of nystatin on fungal infection with *Rhizopus stolonifer* until lethal concentration effect is reached

**Fig. 8:**
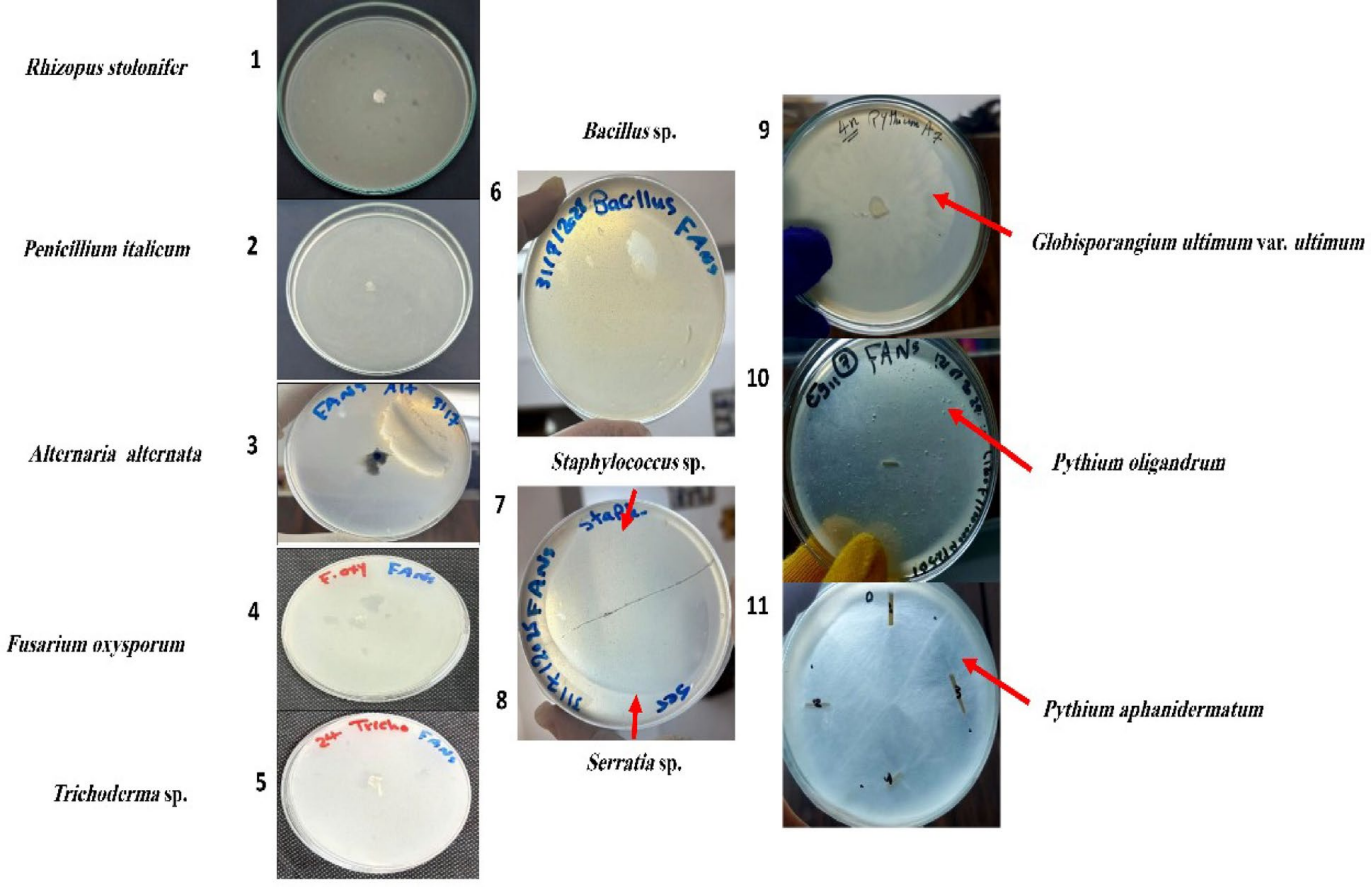
FANS medium (Fluconazole + Ampicillin + Nystatin + Sulbactam) was tested against five fungal species (1–5) and three bacterial species (6–8), and it showed complete inhibitory effect against all tested fungal and bacterial species, while allowing for the isolation and growth of *Globisporangium* (9) and two species of *Pythium* (10, 11)

FANS medium exhibits a complete inhibitory effect against the tested microorganisms that may cause contamination in natural environments (e.g., three bacterial species such as *Bacillus* sp., *Serratia* sp. and *Staphylococcus* sp.). In addition, two other filamentous fungi like *Fusarium oxysporum* and *Trichoderma* sp. Thus, confirming its effectiveness as a selective growth medium for Pythiaceous species (Oomycota) (Fig. 8)

### Impact of selective media of VP_3_, NARF, NARM, and FANS on P. aphanidermatum, P. oligandrum, and G. ultimum var. ultimum mycelial growth

The results showed the highest fungal growth rate at FANS, followed by NARM, then NARF, and finally VP_3_, as shown in Table 4.

**Table 4:** Effect of antibiotics on mycelial growth of three tested *Pythium* species on PDB at 25 °C

Selective media	Dry weight (mg/50 ml)		
	*P*. *aphanidermatum*	*P*. *oligandrum*	G. ultimum var. ultimum
Control (Without antibiotics)	122 ± 1	94 ± 1	112 ± 2
FANS (Fluconazol +=Ampicillin +Nystatin +Sulbactam)	120 ± 3	95 ± 1	110 ± 4
VP_3_ (Vancomycin + PCNB + Penicillin +Pimaricin)	75** ± 3	59** ± 2	77** ± 2
NARM (Nystatin + Ampicillin + Rifampicin + Miconazole)	101* ± 4	90 ± 1	101* ± 3
NARF (Nystatin + Ampicillin + Rifampicin + Fluazinam)	100* ± 2	88* ± 3	96** ± 2

The data consist of the standard error plus the mean of six readings. **Numbers represent differences between sample averages and the control sample when compared to the control, significant values indicate: * = moderately significant at *p* < 0.05, ** = very significant at *p* < 0.01

### Impact of VP_3_, NARF, NARM, and FANS selective media on P. aphanidermatum and P. oligandrum zoospore formation

**Fig. 9:**
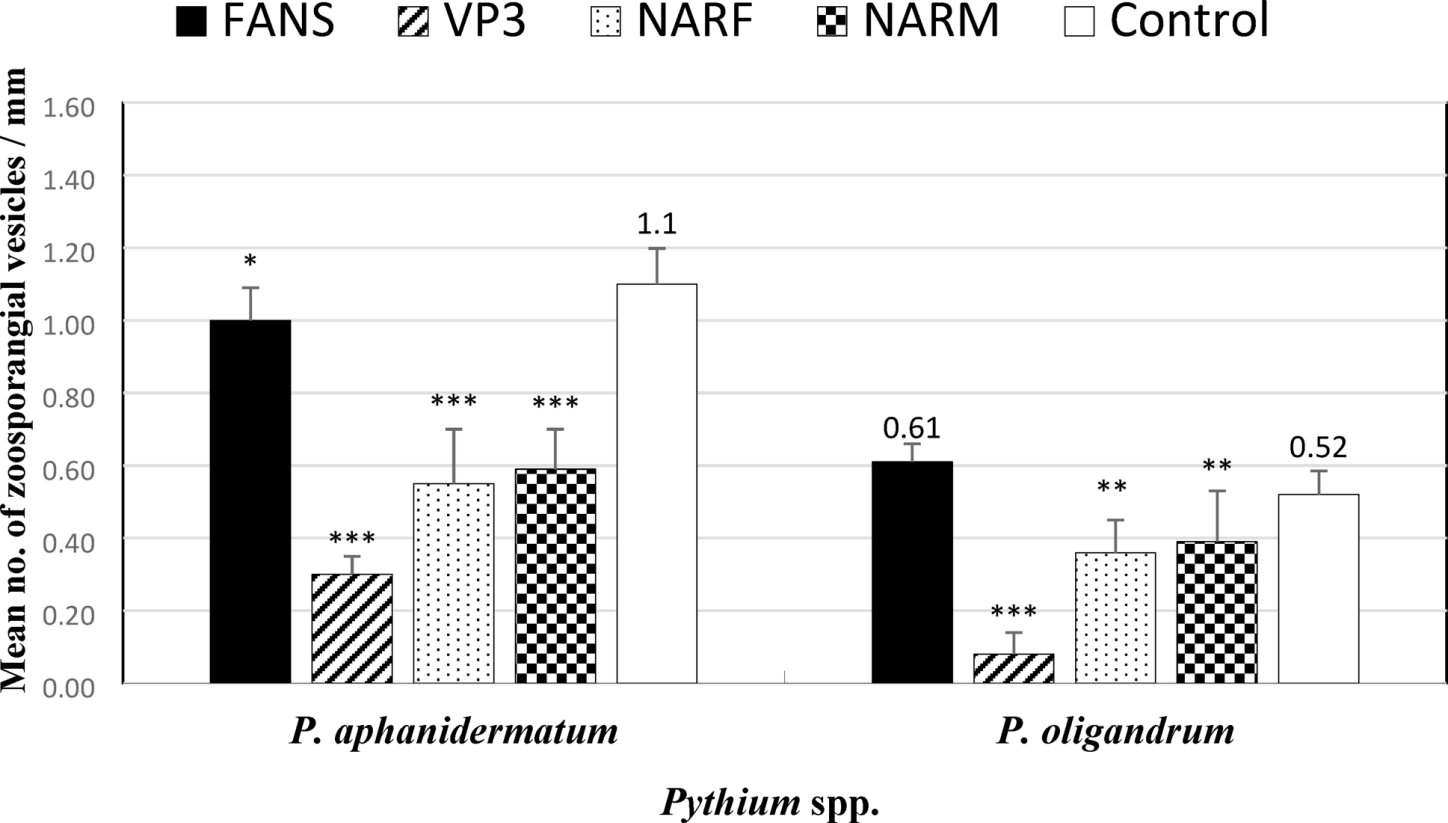
Effect of types of selective (FANS, VP_3_, NARF and NARM) media on zoospore production by *Pythium aphanidermatum* and *Pythium oligandrum* at 25 °C. Data are averages [± S.E. (error bars)] of 5 replicates and significant values against control represent: * = moderately significant at *p* < 0.05, ** = highly significant at *p* < 0.01, *** = very significant at p< 0.001

Compared to the production of zoospores in the control sample, the *Pythium* spp. used in this experiment produced the highest numbers of zoospores when the aqueous medium was treated with antibiotics used in the FANS medium compared to the NARF and NARM mediums, while the VP_3_ medium gave the lowest numbers (Fig. 9).

### Impact of VP_3_, NARF, NARM, and FANS selective media on P. aphanidermatum, P. oligandrum, and G. ultimum var. ultimum oospore production

**Fig. 10:**
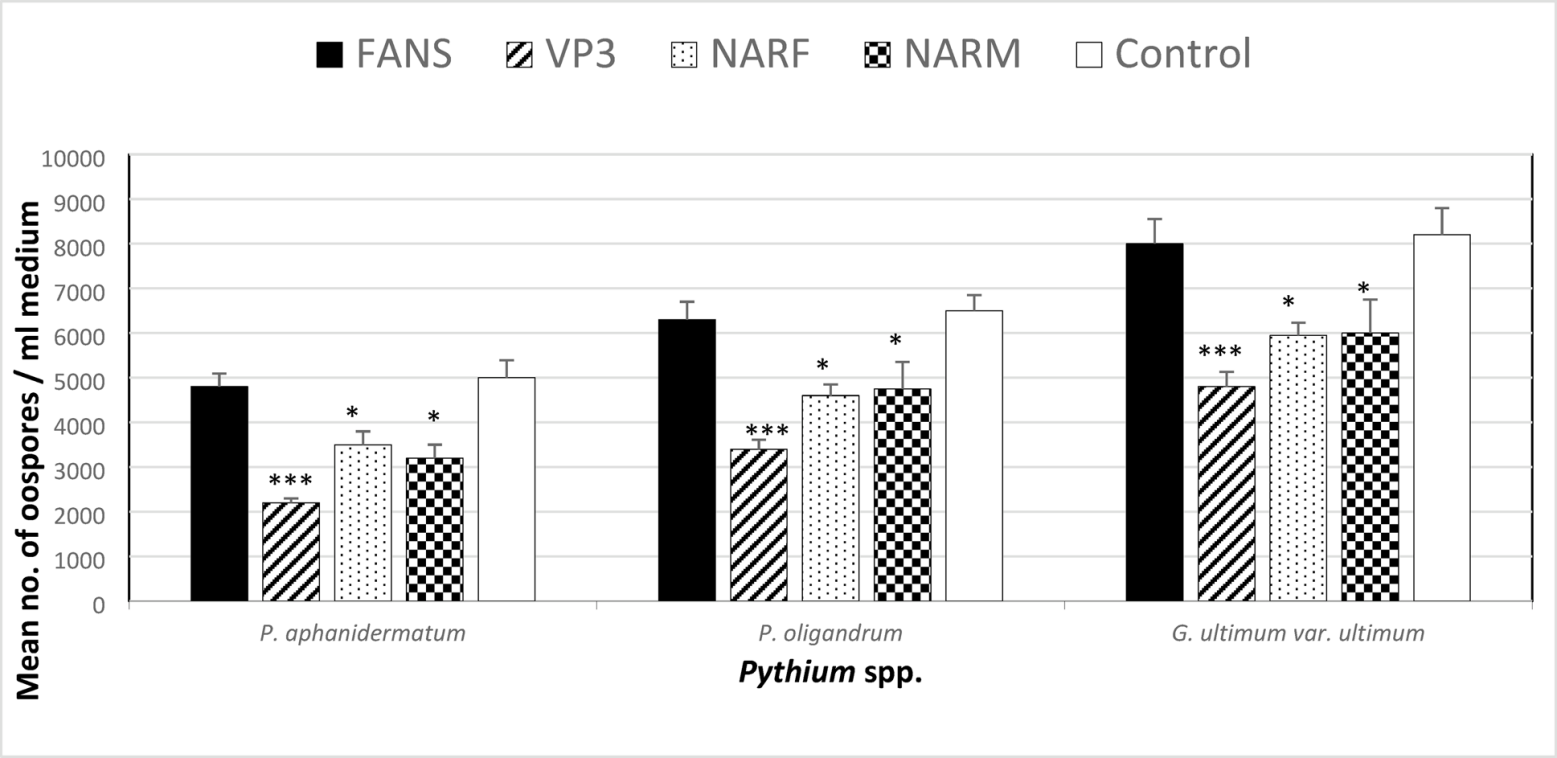
Effect of types of selective (FANS, VP_3_, NARF and NARM) media on oospore production by *Pythium aphanidermatum*, *Pythium oligandrum* and *Globisporangium ultimum* var. *ultimum* at 25 °C. Data are averages [± S.E. (error bars)] of 5 replicates and significant values against control represent: * = moderately significant at *p* < 0.05, ** = highly significant at *p* < 0.01, *** = very significant at *p* < 0.001

When *Pythium* spp. and *G. ultimum* var. *ultimum* were grown in various selective media, *Pythium* spp. employed in this experiment produced varying amounts of oospores in comparison to the production in the control sample. Several antibiotics were used to treat these media. The effect of antibiotics utilized in the VP_3_, NARF, and NARM media were contrasted with those used in the FANS medium. VP_3_ medium produced the fewest oospores, while the FANS medium produced the most, followed by NARF and NARM media **(**Fig. 10).

## Discussion

One of the basics of isolating specific species of fungi from different environmental sources is the use of selective nutrient media that are compatible with a specific fungus that is not affected by the presence of selected types of antibiotics. The physiological effect of certain types of antibiotics on a particular group of fungi is largely attributed to the composition of the cell wall of the organisms being treated. An antifungal drug that interacts with a fungal target that is absent from other eukaryotic cells has been created that is selectively lethal. The fungal cell’s manufacture of crucial structural components is selectively inhibited as part of this tactic. One such therapeutic target is the fungal cell wall. Furthermore, antibiotics that prevent the fungus cell from growing have been found. Glucan synthesis is blocked by echinocandin lipopeptides and papulacandins, chitin synthesis is hindered by polyoxins and nikkomycins, and mannan is selectively bound by pradimicins (Debono and Gordee 1994). Many previous studies have shown the chemical composition of the cell walls of *Pythium* as Up to 38% of a somewhat branched, storage (1,3), (1,6) beta-D-glucan was present in the mycelium. The mycelium’s cell-wall polysaccharides were composed of 82% (1,3), (1,6) beta-D-glucans and 18% cellulose (Blaschek et al. 1992). In summary, we can say that two key characteristics set Oomycota apart from fungi: the makeup of their cell walls and membranes. The Oomycota cell wall typically lacks chitin, a crucial component of the fungal cell wall. Ergosterol is not a major sterol in the cell membrane of Oomycota, which is another way that they vary from fungi (Washabau and Day 2012).

The antibiotics added to the culture medium developed in this study (FANS) consisted of two antibacterial substances (Sulbactam + Ampicillin) and two antifungal substances (Nystatin + Fluconazole). Nystatin is a topical polyene antifungal that binds to sterols in fungal cells. Nystatin is an antifungal antibiotic generated by *Streptomyces noursei*. Nystatin exerts its antifungal effect by attaching to sterols in the fungal cell membrane. Since bacterial and mammalian cells lack sterols in their cell membranes, the medication is ineffective against them. The membrane can no longer serve as a selective barrier to stop potassium and other cellular components from leaving the fungal cell as a result of this interaction. Nystatin exhibits fungistatic or fungicidal properties against a range of yeast and fungal strains, both pathogenic and non-pathogenic (Gallagher et al. 2007).

The second antifungal used here is fluconazole. The fungal cell membrane is changed by fluconazole. Though the specific inhibition of 14-alpha demethylase, it produces its fungistatic activity. This cytochrome P-450 enzyme is essential for the synthesis of ergosterol, a crucial part of the fungal cell membrane. Lanosterol is converted into ergosterol by 14-alpha demethylase. In the absence of ergosterol, the levels of 14 alpha-methyl sterols rise, leading to an increase in cellular permeability in the fungal cell membrane. As a result, the cell’s internal components leak (Sorgo et al. 2011).

The first antibacterial tested here is Ampicillin. It is a semi-synthetic antibiotic with broad spectrum activity that is produced by adding various side chains to the penicillin nucleus. It has a broad-spectrum penicillin, interferes with bacterial cell-wall synthesis during active replication, causing bactericidal activity against susceptible organisms. It demonstrated effectiveness against both Gram-positive and Gram-negative bacteria, but it was more effective against Gram-negative bacteria due to its greater capacity to pierce their outer membrane (Tarannum et al. 2020).

The second antibacterial tested here is sulbactam. Sulbactam is a semisynthetic beta-lactamase inhibitor which when combined with certain beta-lactam antibacterials extends their activity against bacteria that are normally resistant to the antibiotic due to production of beta-lactamases. In combination with ampicillin, it extends the antibacterial activity of ampicillin to include beta-lactamase-producing strains which are otherwise resistant, including *Bacteroides fragilis*, and increases the susceptibility of many sensitive strains (Campoli-Richards and Brogden 1987).

This cocktail, which was selected in the selective nutrient medium (FANS), has been confirmed in many previous studies as antifungal and antibacterial agents. Although some pharmacological precautions on the use of antifungals were used in previous selective media to isolate *Pythium* spp., the antibiotics in FANS are safe as they are used as drugs for human treatment. In adding to the ease of obtaining them from anywhere in the world, in addition to their low price compared to laboratory chemicals.

When the efficiency of the culture media used before this study (VP_3_, NARM and NARF) in isolating *Pythium* spp. was compared with the culture medium developed here (FANS), the results proved that FANS was significantly superior in mycelial growth, zoospore and oospore production for three *Pythium* spp., namely *P. aphanidermatum*, *P. oligandrum* and *G. ultimum* var. *ultimum.*

Based on the logical arguments emerging from the results of this research, we can strongly assert the suitability of using FANS as a selective culture medium for isolating *Pythium* spp. from their natural sources. This will facilitate researchers in the field of Pythiaceous (Oomycota) to study this important genus in the field of academic and applied study in plant diseases. Consequently, the development of affordable microbiological tools, such as developing a new selective medium as in this study, is a crucial component of circular bioeconomy and sustainable biotechnology initiatives, especially when it comes to disease control in environments with limited resources. Notably, the newly developed FANS selective medium exhibited remarkable efficacy in selectively isolating *Pythium* spp., while completely inhibited the growth of other contaminated fungi and bacteria, thus facilitating the characterization morphologically, which represent a start point for future studies such as molecular characterization using ITS region and coxI markers that will confirm their identification and taxonomy in addition to their ecological roles.

Interestingly, FANS medium not only supports the recovery of pathogenic fungi but also potentially beneficial antagonists, making it a promising tool for both biodiversity studies and the development of biological control strategies. The developing FANS medium aligns with modern sustainable biotechnology trends by enabling selective, low-cost recovery of *Pythium* using low-cost, safer and available substrates. This approach is supported by recent studies, such as cultivating *P. irregulare* on food-industry by-products to produce EPA (Russo et al. 2023). Another study developed SMART method, a novel approach to design selective media that combine a specific carbon source and antimicrobials to highly suppress unwanted microbes while selectively support the growth of target bacterial species. The media also support practical and sensitive detection techniques (Sakai et al. 2011).

## Data Availability

No datasets were generated or analysed during the current study.

## Abbreviations

FANS: Fluconazole + Ampicillin + Nystatin + Sulbactam
VP_3_: Vancomycin + PCNB + Penicillin + Pimaricin
NARM: Nystatin + Ampicillin + Rifampicin + Miconazole
NARF: Nystatin + Ampicillin + Rifampicin + Fluazinam
CF: Cystic fibrosis
PCNB: Pentachloronitrobenzene
HCB: Hexachlorobenzene
CMA: Corn Meal Agar
DW: Distilled water
DMSO: Dimethyl sulfoxide
*P. italicum*: *Penicillium italicum*
*R. stolonifer*: *Rhizopus stolonifer*
*P. aphanidermatum*: *Pythium aphanidermatum*
*P. oligandrum*: * Pythium oligandrum*
*G. ultimum* var. *ultimum*: *Globisporangium ultimum* var. *ultimum*
*E. colonum*: * Echinochloa colonum*
V-8 juice: 8 vegetable juice

## References

[CR2] Abdelzaher HMA, Moustafa SMN, Al-Sheikh H (2020) The genus *Pythium* in three different continents. In: Rai M, Abd-Elsalam KA, Ingle AP (eds) *Pythium*: diagnosis, diseases and management. CRC, Boca Raton, pp 15–29. 10.1201/9780429296406.

[CR1] Ali-Shtayeh MS, Len C, Lim-Ho, Dick MW (1986) An improved method and medium for quantitative estimates of populations of *Pythium* species from soil. Trans Br Mycol Soc 86(1):39–47. 10.1016/S0007-1536(86)80116-4

[CR3] Blaschek W, Käsbauer J, Kraus J, Franz G (1992) Pythium aphanidermatum: culture, cell-wall composition, and isolation and structure of antitumour storage and solubilised cell-wall (1–3), (1–6)-beta-D-glucans. Carbohydr Res 231:293–307. 10.1016/0008-6215(92)84026-O 1394320 10.1016/0008-6215(92)84026-o

[CR4] Burr TJ, Stanghellini ME (1973) Propagule nature and density of *Pythium aphanidermatum* in field soil. Phytopathology 63:1499–1501

[CR5] Campoli-Richards DM, Brogden RN (1987) Sulbactam/ampicillin: a review of its antibacterial activity, pharmacokinetic properties, and therapeutic. Drugs 33(6):577–609. 10.2165/00003495-198733060-00003 3038500 10.2165/00003495-198733060-00003

[CR6] Choudhury H, Coleman J, Mink FL, De Rosa CT, Stara JF (1987) Health and environmental effects profile for pentachloronitrobenzene. Toxicol Ind Health 3(1):5–69. 10.1177/074823378700300102 3590208 10.1177/074823378700300102

[CR7] Debono M, Gordee RS (1994) Antibiotics that inhibit fungal cell wall development. Annu Rev Microbiol 48:471–497 7826015 10.1146/annurev.mi.48.100194.002351

[CR8] Dick MW (1990) Keys to *Pythium*. College of estate management. Whiteknights, Reading, p 64

[CR9] Eckert JW, Tsao PH (1962) A selective antibiotic medium for isolation of *Phytophthora* and *Pythium* from plant roots. Phytopathology 52(8):71–777

[CR10] Elnaghy MA, Fadl-Allah EM, Abdelzaher HMA, Moharam SA (2010) Some chemical and physical factors affecting oospores production and germination of wild species of *Pythium irregulare*, *Pythium longisporangium* and *Pythium Oligandrum*. Bull Fac Sci Assiut Univ 39:1–11

[CR11] Friends of the Earth (2024) Environmental and economic risks of pesticide use in agriculture: towards sustainable alternatives. Environ Policy Rep 15(4):45–58

[CR12] Gallagher JG, Williams-Bouyer N, Villarreal C, Heggers JP, Herndon DN (2007) Chap. 12—treatment of infection in burns. In: Herndon DN (ed) Total burn care, 3rd edn. W.B. Saunders, pp 136–176

[CR13] Harvey JV (1925) A study of the water molds and *pythiums* occurring in the soil of chapel hill. J Elisha Mitchell Scient Soc 41:151–164

[CR45] https://wiki.bugwood.org/Diagnosticians_cookbook. Accessed 7 Oct 2024

[CR14] Hu L, Huang X, Yee NH, Meng H, Jiang L, Liang L, Chen X (2024) *Pythium insidiosum*: an emerging pathogen that is easily misdiagnosed and given treatment as a fungus. Front Cell Infect Microbiol 14:1430032. 10.3389/fcimb.2024.1430032 39268488 10.3389/fcimb.2024.1430032 PMC11390559

[CR15] Jayaraj J, Cumagun CJR, Ito H, Arora DK (2006) Selective media and methods for isolation and study of *Pythium* spp. Mycol Res 110(3):243–255. 10.1016/j.mycres.2005.12.004

[CR16] Jeffers SN, Martin SB (1986) Comparison of two media selective for *Phytophthora* and *Pythium* species. Plant Dis 70:1038–1043. 10.1094/PD-70-1038

[CR17] KrajaejunT, Yolanda H, Jearawuttanakul K, Wannalo W, Kanjanasirirat P, Borwornpinyo S,Rujirawat T, Payattikul P, Kittichotirat W, Wichadakul D (2024) Potential anti-Pythiuminsidiosum therapeutics identified through screening of agriculturalfungicides. MicrobiolSpectr12:e01620-23. 10.1128/spectrum.01620-23. PMC10846074 38179943

[CR46] Kipngeno PK, Njuguna JT, Cheruiyot EK (2015) Reduced sensitivity of *Pythium* species to fungicides and implications for disease control. Crop Prot 74:60–66. 10.1016/j.cropro.2015.03.010

[CR20] Lamichhane, J. R., Dürr, C., Schwanck, A. A.,Robin, M. H., Sarthou, J. P., Cellier, V., ... & Aubertot, J. N (2017) Integrated management of damping-off diseases. A review. Agron Sustain Dev 37(2):10. ‏https://doi.org/10.1007/s13593-017-0417-y.

[CR47] Lee D (2023) Storage & Revival of Oomycetes. protocols.io. 10.17504/protocols.io.n92ldmqw7l5b/v1

[CR19] Lodhi AM, Shahzad SALEEM, A. B. D. U. L G (2004) Re-description of pythium adhaerens sparrow. Pak J Bot 36(2):453–456

[CR21] Middleton JT (1943) The taxonomy, host range and geographic distribution of the genus *Pythium*. Mem Torrey Bot Club 20(1):1–171. http://www.jstor.org/stable/43390608

[CR22] Morita Y, Tojo M (2007) Modifications of PARP medium using fluazinam, miconazole, and Nystatin for detection of *Pythium* spp. In soil. Plant Dis 91(12):1591–1599. 10.1094/PDIS-91-12-1591 30780596 10.1094/PDIS-91-12-1591

[CR18] Nguyen D, Vilela R, Miraglia BM, Vilela G, Jasem-Alali N, Rohn R, Mendoza L (2022) Geographic distribution of *Pythium insidiosum* infections in the united States. J Am Vet Med Assoc 260(5):530–534 10.2460/javma.20.10.0595 34968184

[CR23] Oudemans PV (1999) *Phytophthora* species associated with cranberry root rot and surface irrigation water in new Jersey. Plant Dis 83:251–258. 10.1094/pdis.1999.83.3.251 30845503 10.1094/PDIS.1999.83.3.251

[CR24] Özgen M, Kilic K (2025) Molecular techniques for *Pythium* species identification and population diversity analysis. Turk J Agric Food Sci Technol. https://agrifoodscience.com/index.php/TURJAF/article/view/7613

[CR26] Pringsheim N (1858) Beitrage Zur morphologie und systematik der algen 2. Die saprolegnieen. Jb Wiss Bot 1:284–306. 10.5962/bhl.title.60239

[CR31] Radice M et al (2024) Equine pythiosis: an overview. Today’s veterinary Nurse. https://todaysveterinarynurse.com/equine-medicine/equine-pythiosis-an-overview/

[CR29] Rasgele PG, Kaymak F (2013) Evaluation of genotoxic and cytotoxic effects of Natamycin in mice bone marrow cells. Pakistan J Zool 45(4):1103–1112

[CR28] Rencüzoğullari E, Azirak S, Canimoglu S, Parlak S, Buyukleyla M (2009) Effects of Natamycin on sister chromatid exchanges, chromosome aberrations and micronucleus in human lymphocytes. Drug Chem Toxicol 32(1):47–52. 10.1080/01480540802431371 19514938 10.1080/01480540802431371

[CR27] Robertson GI (1980) The genus *Pythium* in new Zealand. N Z J Bot 18(1):73–102. 10.1080/0028825X.1980.10427234

[CR30] Russo GL, Langellotti AL, Martín-García B, Verardo V, Romano R, Sacchi R, Masi P (2023) New biotechnological production of EPA by *Pythium irregulare* using alternative sustainable media obtained from food industry by-products and waste. Sustainability 15(2):1147. 10.3390/su15021147

[CR33] Sakai H, Nagano Y, Sagemoto K, Takai K, Terada T (2011) Selective medium-design algorithm restricted by two constraints (SMART): a novel strategy for designing highly selective media for isolation of target bacteria. PLoS ONE 6(2):e16512. 10.1371/journal.pone.0016512 21304596 10.1371/journal.pone.0016512 PMC3029383

[CR32] Saleem S, Dick MW (1990) Effect of different natural baits on zoospore and oospore production by two *Pythium* species. Pak J Bot 22:125–128

[CR34] Sorgo AG, Heilmann CJ, Dekker HL, Bekker M, Brul S, de Koster CG, de Koning LJ, Klis FM (2011) Effects of fluconazole on the secretome, the wall proteome, and wall integrity of the clinical fungus *Candida albicans*. Eukaryot Cell 10(8):1071–1081. 10.1128/ec.05011-11 21622905 10.1128/EC.05011-11 PMC3165447

[CR35] Tarannum N, Khatoon S, Dzantiev BB (2020) Perspective and application of molecular imprinting approach for antibiotic detection in food and environmental samples: a critical review. Food Control 118:107381. 10.1016/j.foodcont.2020.107381

[CR36] Thambugala KM, Hyde KD, Xu J, Jones EBG (2020) Resistance development in *Pythium* populations to commonly used fungicides: a growing challenge for plant disease management. Plant Pathol 69(9):1544–1552. 10.1111/ppa.13236

[CR37] Tojo M (2017) Selective media for practical isolations of *Pythium* spp. From natural and agricultural environments. Agri Res Tech: Open Access J 7(5):555–723. 10.19080/ARTOAJ.2017.07.555723

[CR25] Van der Plaats-Niterink AJ (1981) Monograph of the genus *Pythium*. Studies in Mycology, vol 21. Baarn, Centraalbureau voor Schimmelcultures, Netherlands, p 242

[CR38] Wang Y et al (2020) Common strategies to control *Pythium* disease. J-Stage. https://www.jstage.jst.go.jp/article/ras/8/0/8_58/_article

[CR39] Washabau RJ, Day MJ (2012) Canine and feline gastroenterology. Elsevier Health Sciences

[CR40] Watanabe H, Kageyama K, Taguchi Y, Horinouchi H, Hyakumachi M (2008) Bait method to detect *Pythium* species that grow at high temperatures in hydroponic solutions. J Gen Plant Pathol 74(6):417–424

[CR41] Waterhouse GM (1968) The genus *Pythium* pringsheim. Diagnoses (or descriptions) and figures from the original papers. Mycological Papers 110:71

[CR42] Yang Y, Zhang J, Yan J, Zhao L, Luo L, Li C, Yang G (2024) Effects of chemical and biological fungicide applications on sexual sporulation of *Rhizoctonia Solani* AG-3 TB on tobacco. Life 14(3):404. 10.3390/life14030404 38541728 10.3390/life14030404 PMC10971793

[CR43] Yuriko N, Cherie B, Colin M, Goldsmith E, Walker JM, Stuart J, Jackie E, R. and, John EM (2008) Development of selective media for the isolation of yeasts and filamentous fungi from the sputum of adult patients with cystic fibrosis (CF). J Cyst Fibros 7(6):566–572. 10.1016/j.jcf.2008.06.007 18723404 10.1016/j.jcf.2008.06.007

[CR44] Zhang J, Debets AJM, Verweij PE, Schoustra SE (2021) Selective flamingo medium for the isolation of *Aspergillus fumigatus*. Microorganisms 9(6):1155. 10.3390/microorganisms9061155 34072240 10.3390/microorganisms9061155 PMC8228204

